# Defining the Effects of PKC Modulator HIV Latency-Reversing Agents on Natural Killer Cells

**DOI:** 10.20411/pai.v9i1.673

**Published:** 2024-04-24

**Authors:** Melanie Dimapasoc, Jose A. Moran, Steve W. Cole, Alok Ranjan, Rami Hourani, Jocelyn T. Kim, Paul A. Wender, Matthew D. Marsden, Jerome A. Zack

**Affiliations:** 1 Molecular Biology Institute, University of California Los Angeles, Los Angeles, California; 2 Department of Microbiology, Immunology, and Molecular Genetics, University of California Los Angeles, Los Angeles, California; 3 Department of Microbiology and Molecular Genetics, School of Medicine, University of California Irvine, California; 4 UCLA Department of Psychiatry & Biobehavioral Sciences, David Geffen School of Medicine at UCLA, Los Angeles, California; 5 Department of Chemistry, Stanford University, Stanford, California; 6 Department of Medicine, Division of Infectious Diseases, University of California Los Angeles, Los Angeles, California; 7 Department of Chemical and Systems Biology, Stanford University, Stanford, California; 8 Department of Medicine, Division of Infectious Diseases, School of Medicine, University of California, Irvine, Irvine, California; 9 Department of Medicine, Division of Hematology and Oncology, University of California Los Angeles, Los Angeles, California

**Keywords:** Killer Cells, Natural, Protein Kinase C, HIV-1, Virus Latency, Immunity, Acquired Immunodeficiency Syndrome

## Abstract

**Background:**

Latency reversing agents (LRAs) such as protein kinase C (PKC) modulators can reduce rebound-competent HIV reservoirs in small animal models. Furthermore, administration of natural killer (NK) cells following LRA treatment improves this reservoir reduction. It is currently unknown why the combination of a PKC modulator and NK cells is so potent and whether exposure to PKC modulators may augment NK cell function in some way.

**Methods:**

Primary human NK cells were treated with PKC modulators (bryostatin-1, prostratin, or the designed, synthetic bryostatin-1 analog SUW133), and evaluated by examining expression of activation markers by flow cytometry, analyzing transcriptomic profiles by RNA sequencing, measuring cytotoxicity by co-culturing with K562 cells, assessing cytokine production by Luminex assay, and examining the ability of cytokines and secreted factors to independently reverse HIV latency by co-culturing with Jurkat-Latency (J-Lat) cells.

**Results:**

PKC modulators increased expression of proteins involved in NK cell activation. Transcriptomic profiles from PKC-treated NK cells displayed signatures of cellular activation and enrichment of genes associated with the NFκB pathway. NK cell cytotoxicity was unaffected by prostratin but significantly decreased by bryostatin-1 and SUW133. Cytokines from PKC-stimulated NK cells did not induce latency reversal in J-Lat cell lines.

**Conclusions:**

Although PKC modulators have some significant effects on NK cells, their contribution in “kick and kill” strategies is likely due to upregulating HIV expression in CD4^+^ T cells, not directly enhancing the effector functions of NK cells. This suggests that PKC modulators are primarily augmenting the “kick” rather than the “kill” arm of this HIV cure approach.

## INTRODUCTION

Since the start of the HIV epidemic, more than 85 million people have been infected with HIV, and nearly half have died of AIDS-related illnesses [1]. Despite advances in the development of antiretroviral therapy (ART), HIV remains a significant cause of worldwide morbidity and mortality. ART often suppresses HIV to undetectable levels in the plasma; however, a subset of long-lived, latent memory CD4^+^ T cells harboring integrated replication-competent provirus persists and can produce virus upon interruption of therapy, leading to viral rebound [2–5]. Moreover, there are several limitations to lifelong ART, including cost, compliance, the development of drug-resistant viruses [6], and inadequate access to treatment, especially in more resource-limited countries [7], which underscores the need to develop more effective therapeutic approaches beyond lifelong ART.

Significant progress has been made in the development of various cure strategies that target HIV. These approaches include bone marrow transplantation using CCR5-deficient cells [8–10], gene-editing strategies that excise [11–14] or permanently inactivate latent provirus [15], improvement of immune responses [16–22], and elimination of infected cells through other mechanisms [23–25]. One approach, known as the “kick and kill” strategy, focuses on eliminating or reducing the size of the latent reservoir through induction of, or “kicking,” latently infected cells with latency-reversing agents (LRAs) that stimulate expression of viral proteins, thereby allowing them to be targeted or “killed” by virus-induced cytopathic effects, immune clearance, apoptosis, or antiviral therapies [26, 27]. This treatment would be performed in the presence of ART to prevent HIV replication and further spread of infection. Several structural classes of LRAs belonging to distinct functional categories have been reported and have shown efficacy in reversing latency *in vitro, ex vivo*, and in animal models [28–30]. One of the most promising classes of LRAs is the protein kinase C (PKC) modulators [31–40]. PKC modulators activate and recruit the transcription factor NF-kappa B (NFκB), which binds to the HIV LTR to promote transcription [41]. Previous reports have also shown that PKC modulators can affect NK cell functions, including activation, cytotoxicity, antibody-dependent cellular cytotoxicity (ADCC), and secretion of interferon-gamma (IFNγ), an important pro-inflammatory cytokine involved in the pathogenesis of HIV/AIDS [42–44]. The PKC modulator prostratin (Supplemental Figure 1A), a non-tumorigenic phorbol ester derived from the bark of the Samoan medicinal plant *Homalanthus nutans*, has been shown to promote transcriptional activation of latent HIV provirus, as well as inhibit viral replication by down-regulating the HIV entry receptors CD4 and CXCR4 [45–48]. Purified NK cells showed enhanced killing of autologous CD4^+^ T cells harboring reactivated HIV *in vitro* when both cell types were stimulated with prostratin [49]. Another PKC modulator, bryostatin-1 (Supplemental Figure 1B), a naturally occurring macrocyclic lactone derived from the marine invertebrate *Bugula neritina*, has also shown potency in reversing HIV latency and is the only PKC modulator that has been tested in clinical trials in ART-treated individuals [50]. However, results from this study demonstrated that single administration of bryostatin-1 at the low doses tested induced neither PKC activation nor HIV transcription *in vivo*. Moreover, bryostatin-1 inhibited target cell lysis and ADCC function of NK cells [49]. These findings, combined with the issues of a narrow therapeutic window and limitations associated with natural sourcing [51], highlight the need for better latency-reversing compounds.

Recent practical syntheses of the natural PKC modulators prostratin and bryostatin-1 have enabled the creation of designed and synthetic PKC modulator analogs that exhibit superior efficacy and tolerability when compared to their parent compounds [32, 48, 52–56]. One notable bryostatin-1 analog, SUW133 (Supplemental Figure 1C), was shown to reverse HIV latency in cell lines and patient-derived cells [48]. Moreover, *in vivo* treatment with SUW133 prior to cessation of ART reduced barcoded HIV diversity, caused a delay in viral rebound, and reduced the number of rebounding viral lineages in a humanized mouse model of HIV latency, suggesting a reduction in the latent reservoir [57]. Significantly, a greater reduction in the rebound-competent HIV reservoir was observed in similar experiments in which NK cells were also injected into ART-suppressed animals following the administration of SUW133 [58]. While SUW133 resulted in up-regulation of several cytokines during *in vitro* stimulation of primary human peripheral blood mononuclear cells (PBMCs), these secreted factors did not directly induce HIV latency reversal [59], suggesting SUW133's effects in reversing latency are primarily mediated by a direct cellular response to the compound rather than indirect stimulation of latently infected cells via secreted factors and cytokines. These results demonstrate SUW133's potential as a compelling candidate in the pursuit of HIV eradication and that it warrants further characterization of the molecular mechanisms underpinning its effects on kick and kill strategies.

Here, we sought to characterize the effects of PKC modulators, particularly SUW133, on human peripheral NK cells to identify why combining PKC modulators and NK cells is more potent in depleting latently infected cells than a single treatment alone and whether PKC modulators have a direct effect on NK cell function, which may explain this observation. We showed that while PKC modulators activate NK cells and modify their gene expression profile and phenotype, exposure to SUW133 did not enhance NK cell cytotoxic function. Additionally, consistent with previous findings in PBMCs, SUW133 led to increased secretion of cytokines by NK cells, but these secreted factors did not independently induce HIV latency reversal. Most importantly, through transcriptomic analysis, we report that the effects of PKC modulators were more robust in CD4^+^ T cells than in NK cells. Together, these data suggest that the primary impact of PKC modulators, when used in combination with NK cells, lies in stimulating the induction of HIV expression (the kick arm) rather than enhancing the elimination of reactivated cells by improving NK cell cytotoxic activity (the kill arm) of this proposed approach.

## METHODS

### Isolation of NK and CD4^+^ T Cells

De-identified PBMCs from healthy, HIV seronegative human donors were obtained with informed consent from the UCLA AIDS Institute Virology Core Laboratory under IRB approval. Prior to cell isolation, adherent macrophages were removed from PBMCs by culturing in flasks overnight in R10 media (RPMI 1640 medium supplemented with 10% vol/vol fetal bovine serum [FBS, Omega Scientific] and 1% penicillin/streptomycin [Invitrogen]). Primary NK and resting CD4^+^ T cells were isolated from PBMCs by negative selection using the NK Isolation Kit (Miltenyi Biotec) and EasySep Human Resting CD4^+^ T Cell Isolation Kit (STEMCELL Technologies), respectively, according to the manufacturers' protocols. Purity of isolated NK cells was evaluated by staining with Ghost Violet 510 (Tonbo Biosciences); CD3-Alexa Fluor 700 (A700, clone HIT3a, BioLegend); CD4-Allophycocyanin-Cyanine7 (APC-Cy7, clone OKT4, BioLegend); CD8-Peridinin-Chlorophyll-Protein-Cyanine5.5 (PerCP-Cy5.5, clone RPA-T8, BioLegend); CD14-Phycoerythrin (PE, clone M5E2, BioLegend); CD19-PE-Cy7 (clone HIB19, BioLegend); CD45-Pacific Blue (PB, clone HI30, BioLegend); CD56-APC (clone 5.1H11, BioLegend); and CD69-Fluorescein (FITC, clone FN50, BioLegend). Resting CD4^+^ T cells were assessed for purity by staining with Ghost Violet 510 (Tonbo Biosciences); CD3-A700 (clone HIT3a, BioLegend); CD4-APC-Cy7 (clone OKT4, BioLegend); CD8-PerCP-Cy5.5 (clone RPA-T8, BioLegend); CD14-PE (clone M5E2, BioLegend); CD25-APC (clone M-A251, BioLegend); CD45-PB (clone HI30, BioLegend); CD69-FITC (clone FN50, BioLegend); and HLA-DR-PE-Cy7 (clone LN3, BioLegend). Data was collected on an LSR Fortessa flow cytometer (BD Biosciences) and analyzed with FlowJo software (version 10.8.1 or later). Purified NK and resting CD4^+^ T cells were frozen at a concentration of 5x10^6^ cells/mL in Bambanker (GC Lymphotec) and stored in liquid nitrogen prior to use.

### Assessment of NK Cell Activation by Flow Cytometry

NK cells were thawed and cultured overnight in C10 media consisting of RPMI 1640 media supplemented with 10% vol/vol FBS (Omega Scientific), 1% L-glutamine, 1% penicillin/streptomycin (Invitrogen), 500mM 2-mercaptoethanol (Sigma), 1mM sodium pyruvate (Gibco), 0.1mM MEM non-essential amino acids (Gibco), and 10mM HEPES (Gibco). NK cells were then cultured in C10 media containing 10nM bryostatin-1, 1µM prostratin, or 10nM SUW133 for 24 hours at 37°C and 5% CO_2_. Unstimulated cells (DMSO only) were cultured in parallel throughout LRA stimulations in C10 medium. To stain for CD107a (LAMP-1), cell cultures were supplemented with CD107a-APC mAb (clone H4A3, BioLegend) 1 hour after the addition of the compound, then 1X Protein Transport Inhibitor Cocktail (eBioscience) was added and cells were cultured for an additional 23 hours. Cells were then washed twice with phosphate buffered saline (PBS) and resuspended in a 1:1000 dilution of Ghost Violet 510 (Tonbo Biosciences) in PBS at 4°C for 30 minutes. Cells were washed with PBS + 2% FBS and then resuspended in a 1:1 dilution of PBS:Human AB serum (Sigma). To label cell-surface molecules, the following fluorescent conjugated antibodies were then added: CD3-A700 (clone HIT3a, BioLegend); CD4-APC-Cy7 (clone OKT4, BioLegend); CD56-PB (clone 5.1H11, BioLegend); CD69-FITC (clone FN50, BioLegend); and NKG2D-PECy7 (clone 1D11, BioLegend). During staining, cells were incubated at 4°C for 30 minutes, then washed with PBS + 2% FBS. Cells were fixed in 1% paraformaldehyde at room temperature for 20 minutes, then washed and resuspended in PBS + 2% FBS and stored at 4°C until collection. Data was collected on an LSR Fortessa flow cytometer (BD Biosciences) and analyzed with FlowJo software (version 10.8.1 or later). Statistical analyses were performed using GraphPad Prism (version 9.4.1 or later).

### Quantification of Secreted Cytokines

Supernatant samples from LRA-stimulated cells were analyzed by the UCLA Immune Assessment Core using the Luminex 38-plex Human Cytokine/Chemokine panel. For the purpose of statistical analysis, undetectable values were reported as the mean between 0 and the quantification limit, which varies by cytokine. Statistical analyses were performed using GraphPad Prism (version 9.4.1 or later).

### RNA Isolation and Sequencing

Frozen NK and CD4^+^ T cell pellets were submitted to the UCLA Technology Center for Genomics and Bioinformatics (TCGB) for RNA extraction and sequencing. RNA was extracted using an RNeasy mini kit (QIAGEN). Libraries for RNA-seq were prepared with a KAPA mRNA HyperPrep Kit. The workflow consists of the depletion of rRNA by hybridization of complementary DNA oligonucleotides, followed by treatment with RNase H and DNase. The next steps include mRNA enrichment and fragmentation, first-strand cDNA synthesis using random priming followed by second-strand synthesis converting cDNA:RNA hybrid to double-stranded cDNA (dscDNA), and incorporating dUTP into the second cDNA strand. Next, cDNA generation is followed by end repair to generate blunt ends, A-tailing, adaptor ligation, and PCR amplification. Different adaptors were used for multiplexing samples in one lane. Sequencing was performed on Illumina NovaSeq6000 for a paired-end 2x50 run. A data quality check was done on Illumina SAV. Demultiplexing was performed with Illumina Bcl2fastq v2.19.1.403 software. The reads were mapped by STAR 2.7.9a [60], and read counts per gene were quantified using the human genome GRCh38.104. In Partek Flow [61], read counts were normalized by CPM +1.0E-4 to generate PCA plots. For transcriptomic analysis, gene expression data were quantified as gene transcripts per million mapped reads (TPM), with values floored at 1 TPM (to suppress spurious variability) and log_2_ transformed (to stabilize variance) for statistical analysis of differential expression using a standard linear statistical model (base R lm procedure) to quantify the magnitude of differential gene expression across conditions. Venn diagrams were generated using BioVenn [62]. Transcription factor enrichment analysis was performed using Metascape [63].

### NK Cell Cytotoxicity Assays

For the K562 co-culture assays, previously frozen NK cells were thawed and cultured overnight in C10 media, then stimulated with C10 media containing LRA for 24 hours at 37°C and 5% CO_2_. Unstimulated cells (DMSO only) were cultured in parallel in C10 medium. Wells containing only NK cells, only K562 cells, or K562 cells treated with 0.1% Tween 20 served as assay controls. NK cells were labeled with 200nM 5,6-carboxyfluroscein diacetatesuccinimidyl ester (CFSE, eBio-science) for 15 minutes at 37°C, washed twice, then seeded with 25,000 K562 cells at different effect-to-target (E:T) ratios for 4 hours at 37°C and 5% CO_2_. The co-cultured cells were then labeled with 20 µg/mL 7-aminoactinomycin D (7-AAD, Invitrogen) for 20 minutes at room temperature, washed and fixed in 1% paraformaldehyde at room temperature for 20 minutes, then washed and resuspended in PBS + 2% FBS and stored at 4°C until collection. Data was collected on an LSR Fortessa flow cytometer (BD Biosciences) and analyzed with FlowJo software (version 10.8.1 or later). For each E:T ratio, 10,000 target cells (gated as CFSE^-^) were acquired by FACS. The percentage of specific lysis was calculated as follows: 100 x (%7AAD^+^ target cells in sample – basal %7AAD^+^ target cell death, from K562 only control) ÷ (maximum %7AAD^+^ target cell death, from 0.1% Tween 20 treated K562 cells – basal %7AAD^+^ target cell death, from K562 only control). K562 cells (cat# CCL-243) were obtained through the American Type Culture Collection (ATCC). Statistical analyses were performed using GraphPad Prism (version 9.4.1 or later).

For the HIV-infected CD4^+^ T cell co-culture assays, previously frozen NK^-^ cells were thawed and stimulated for 4 days using Dynabeads Human T-Activator CD3/CD28 for T-Cell Expansion and Activation (Gibco) in C10 medium with IL-2. CD4^+^ T cells were isolated from the NK^-^ cells by positive selection using CD4^+^ microbeads (Miltenyi Biotec) according to the manufacturer's protocol. Purity of isolated CD4^+^ T cells was evaluated as described above. CD4^+^ T cells were then infected with barcoded NL-HABC virus (600 ng/10^6^ cells) by spinoculating 1.5x10^6^ cells/mL in virus containing 5 mg/mL of polybrene in a 24-well plate. Plates were centrifuged at 1200*g* for 2 hours at 25°C. After infection, cells were incubated overnight at 37°C and 5% CO_2_. Previously frozen, donor-matched NK cells were thawed and cultured overnight in C10 media, then stimulated with C10 media containing LRA for 24 hours at 37°C and 5% CO_2_. Unstimulated cells (DMSO only) were cultured in parallel in C10 medium. Wells containing only NK cells, only CD4^+^ T cells, or CD4^+^ T cells treated with 1X Cell Activation Cocktail (without Brefeldin A; BioLegend) served as assay controls. NK and CD4^+^ T cells were washed twice, then seeded at an effect-to-target (E:T) ratio of 1:1 (50,000 NK + 50,000 CD4^+^ T cells) for 4 hours at 37°C and 5% CO_2_. The co-cultured cells were labeled with CD3-A700 (clone HIT3a, BioLegend); CD4-APC-Cy7 (clone OKT4, BioLegend); CD56-PB (clone 5.1H11, BioLegend); and Ghost Violet 510 (Tonbo Biosciences), as described above. To detect surface HA expression, cells were first stained with high affinity anti-HA-Biotin (Sigma) at 4°C for 30 minutes, washed, then stained with streptavidin (R-PE conjugate, Invitrogen). After staining, cells were washed and fixed in 1% paraformaldehyde at room temperature for 20 minutes, then washed and resuspended in PBS + 2% FBS and stored at 4°C until collection. Data was collected on an LSR Fortessa flow cytometer (BD Biosciences) and analyzed with FlowJo software (version 10.8.1 or later). For each sample, 10,000 target cells (gated as CD3^+^CD4^+^) were acquired by FACS. The percentage of specific lysis was calculated as follows: 100 x (%HA^+^ target cells in sample – basal %HA^+^ target cell death, from CD4^+^ T cell only control) ÷ (maximum %HA^+^ target cell death, from 1X CAC treated CD4^+^ T cells – basal %HA^+^ target cell death, from CD4^+^ T cell only control). Statistical analyses were performed using GraphPad Prism (version 9.4.1 or later).

### Conditioned Media Synthesis in NK Cells

Previously frozen NK cells were thawed and cultured overnight in C10 media (RPMI 1640 media supplemented with 10% vol/vol FBS (Omega Scientific), 1% L-glutamine, 1% penicillin/streptomycin (Invitrogen), 500mM 2-mercaptoethanol (Sigma), 1 mM sodium pyruvate (Gibco), 0.1mM MEM non-essential amino acids (Gibco), and 10 mM HEPES (Gibco)). NK cells were then plated in C10 media containing 10nM SUW133 or untreated (DMSO only) for 5 hours at 37°C and 5% CO_2_. Five hours post-stimulation, NK cells were washed twice with C10 to remove LRA, then resuspended in fresh C10 and incubated for an additional 24 hours to allow for the accumulation of cytokines and secreted factors. Culture supernatant (or conditioned media, CM) was collected, filtered using a 0.22-µm filter, and stored at -80°C prior to use.

### HIV Latency Reversal Assay

J-Lat A2 and 10.6 cells were cultured in CM and assessed for HIV expression as described previously [59]. J-Lat A2 (cat# 9854) and 10.6 (cat# 9849) were obtained through the NIH HIV Re-agent Program, Division of AIDS, NIAID, NIH from Dr. Eric Verdin.

## RESULTS

### NK Cells are Activated by PKC Modulators

**Figure 1.**
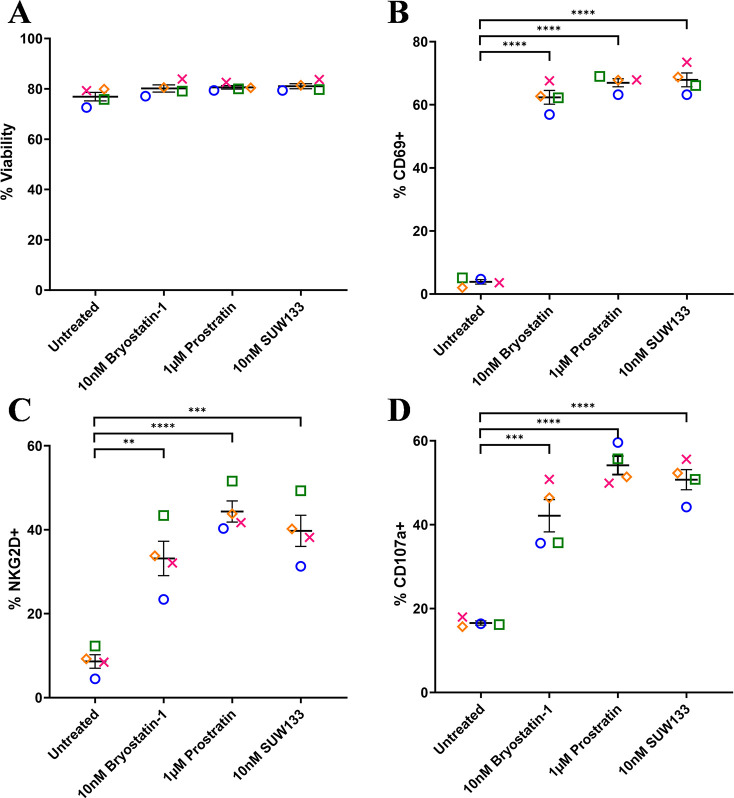
**Viability and cell-surface expression of activation and degranulation markers on NK cells treated with PKC modulators.** NK cells were cultured for 24 hours untreated (DMSO only), with 10nM bryostatin-1, 1μM prostratin, or 10nM SUW133 and analyzed for viability (A), CD69 (B), NKG2D (C), and CD107a (D) via flow cytometry. Data from 4 healthy human donors are shown, with each color and shape representing results from a different donor. Horizontal lines indicate the mean. Error bars indicate the standard error of the mean (SEM). An unpaired, unequal variance Student's *t*-test was performed, with (***) indicating *P* < 0.001 and (****) indicating *P* < 0.0001.

To assess the effect of SUW133 on NK cell viability and degranulation, primary NK cells were isolated from healthy human donors and cultured for 24 hours in various concentrations of SUW133 (Supplemental Figure 2). In all conditions, the viability of NK cells was not significantly affected (Supplemental Figure 2A), thus we decided to use the 10nM concentration of SUW133, which was sufficient to show effects on degranulation (Supplemental Figure 2B) and has been previously shown to reverse HIV-1 latency *in vitro* [35, 38]. For bryostatin-1 and prostratin, concentrations previously shown to reverse HIV-1 latency *in vitro* [32, 34, 35, 38, 41, 65] were used. Primary NK cells were isolated from healthy human donors and cultured for 24 hours in the presence of PKC modulators. After 24 hours of culture, we evaluated NK cell viability, using the cell-surface expression of CD69 and NKG2D to assess the effects of PKC modulators on NK cell activation, and the cell-surface expression of CD107a to assess their effects on NK cell degranulation (Figure 1, Supplemental Figure 3). There was no significant difference in NK cell viability between the different LRA treatment groups and the control (Figure 1A). Consistent with prior work [49], bryostatin-1 and prostratin increased the expression of CD69, NKG2D, and CD107a on NK cells compared to no treatment (DMSO only; Figure 1B-D). Similarly, treatment with SUW133 resulted in increased expression of all 3 markers (Figure 1B-D). Moreover, while there was variation between donors, there was no statistically significant difference in the level of expression of all 3 markers among the 3 PKC modulators. Overall, these results indicate that bryostatin-1, prostratin, and SUW133 induce activation and degranulation in NK cells.

### PKC Modulators Induce Increased Secretion of IFNγ, MIP-1α, MIP-1β, and TNFα by NK Cells

**Figure 2.**
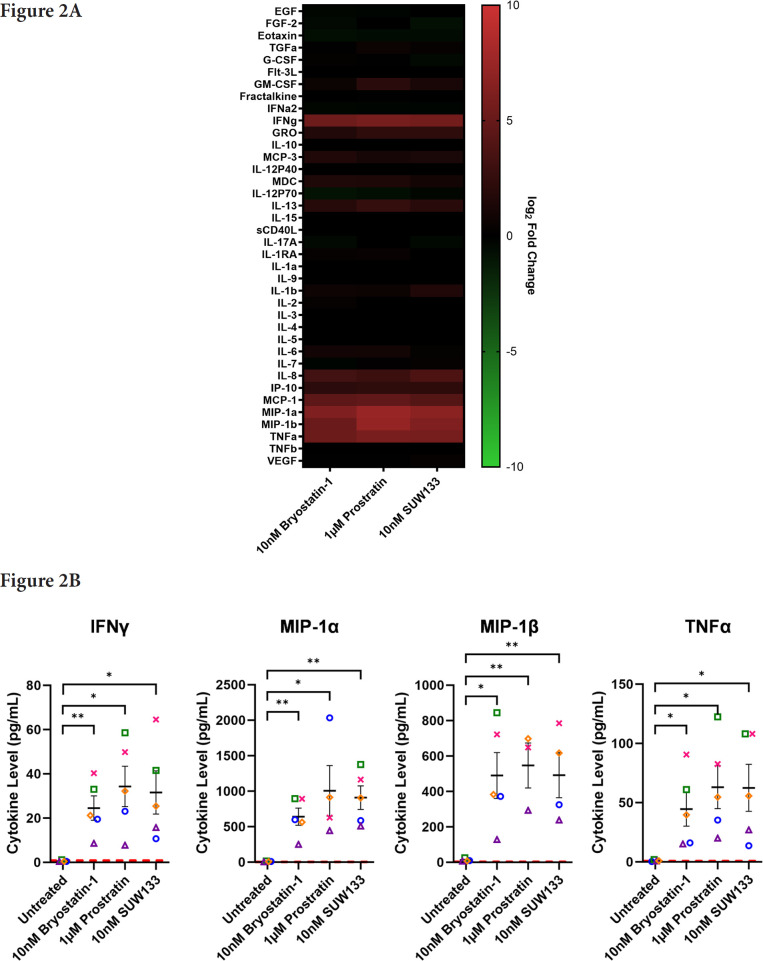
**Cytokine induction in NK cells by PKC modulators.** Supernatant from NK cells treated with 10nM bryostatin-1, 1µM prostratin, or 10nM SUW133 was filtered and analyzed using a Luminex 38-plex human cytokine immunoassay for cytokine composition. (A) Heatmap showing the mean fold change relative to the untreated (DMSO only) control from 5 different biological donors. (B) Example cytokine profiles from data shown in panel A, with each color and shape representing results from a different human donor (n=5). Horizontal lines indicate the mean. Error bars indicate the standard error of the mean (SEM). Dashed red lines indicate the lower limit of detection for the assay, which varies between different cytokines. A 2-tailed, unpaired, unequal variance Student's *t*-test was performed, with (*) indicating *P* < 0.05 and (**) indicating *P* < 0.01

Cytokine composition was analyzed in the supernatant from NK cells treated for 24 hours with PKC modulating LRAs. Consistent with previous studies in PBMCs [48, 52], we observed variations in cytokine concentrations between the 3 conditions, with many cytokines displaying elevated levels compared to the control (Figure 2A). Of the 38 cytokines measured, pro-inflammatory cytokines IFNγ, MIP-1α, MIP-1β, and TNFα showed a significant increase in concentration compared to untreated cells (Figure 2B). IFNγ plays an important role in immune regulation and antiviral response by activating and recruiting cells and inhibiting viral replication [66–71]. MIP-1α and MIP-1β are involved in the development and recruitment of T_H_1 cells [72–74]. These cytokines can also suppress HIV infection by blocking and downregulating cell-surface expression of CCR5 [75–78], which is used as a co-receptor for entry by R5 tropic strains of HIV-1 [79–82]. TNFα is a pleiotropic cytokine that can both stimulate and inhibit HIV replication. It has been shown to activate NFκB and induce HIV transcription [83–88]. However, it can also stimulate the secretion of cytokines that suppress HIV [77, 89–91]. Thus, the secretion of these cytokines by NK cells may play an important role in the immune response against HIV following latency reversal.

### NK and CD4^+^ T Cells Treated with PKC Modulators Show Distinct Gene Expression Profiles

**Figure 3.**
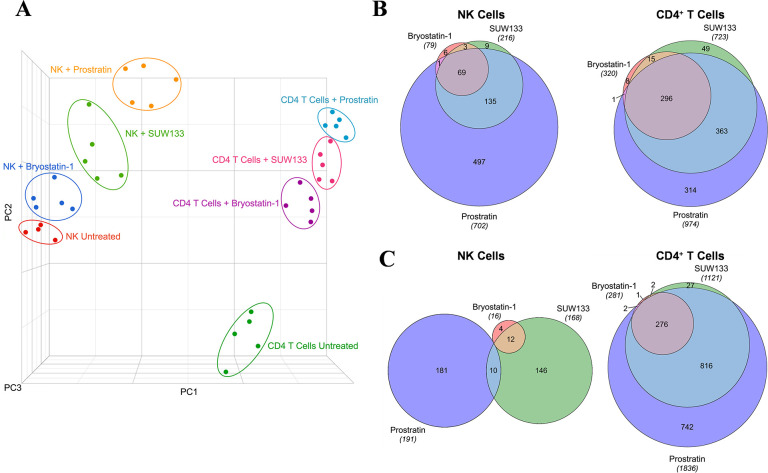
**Transcriptomic profiles of NK and CD4^+^ T cells treated with PKC modulators.** RNA-seq was performed on NK and CD4^+^ T cells cultured for 24 hours untreated (DMSO only), with 10nM bryostatin-1, 1μM prostratin, or 10nM SUW133. (A) Principal component analysis (PCA) plot of data from 5 healthy human donors is shown, with each point representing results from a different donor and each color representing a different treatment group. Venn diagram illustrating the overlap of upregulated (B) and downregulated (C) genes between the 3 different PKC modulators in NK and CD4^+^ T cells. Areas shown are proportional to the numbers of genes within each category.

To define the molecular mechanisms underlying the effects of PKC modulators on NK and CD4^+^ T cells, we performed RNA sequencing (RNA-seq) on LRA-stimulated and untreated cells. The transcriptomic profiles revealed distinct clustering by principal component analysis (PCA) between each condition within both cell types (Figure 3A), indicating unique gene changes induced by bryostatin-1, prostratin, SUW133, and the unstimulated control. Moreover, based on PCA clustering, all conditions for NK cells are more similar to each other than to CD4^+^ T cells and vice versa. Based on the relative distance to the controls, each cell type exhibited the same hierarchy in magnitude of transcriptomic changes between conditions, with prostratin having the greatest effect, followed by SUW133, then bryostatin-1. This was confirmed by comparing the number of differentially expressed genes (DEGs) induced by each PKC modulator (Figure 3B). DEGs were defined as having false discovery rate (FDR) corrected *p*-values (*q*-values) < 0.01 and |log_2_FC| > 2. Prostratin had the greatest number of both up- and down-regulated genes, followed by SUW133, and then bryostatin-1.

**Figure 4.**
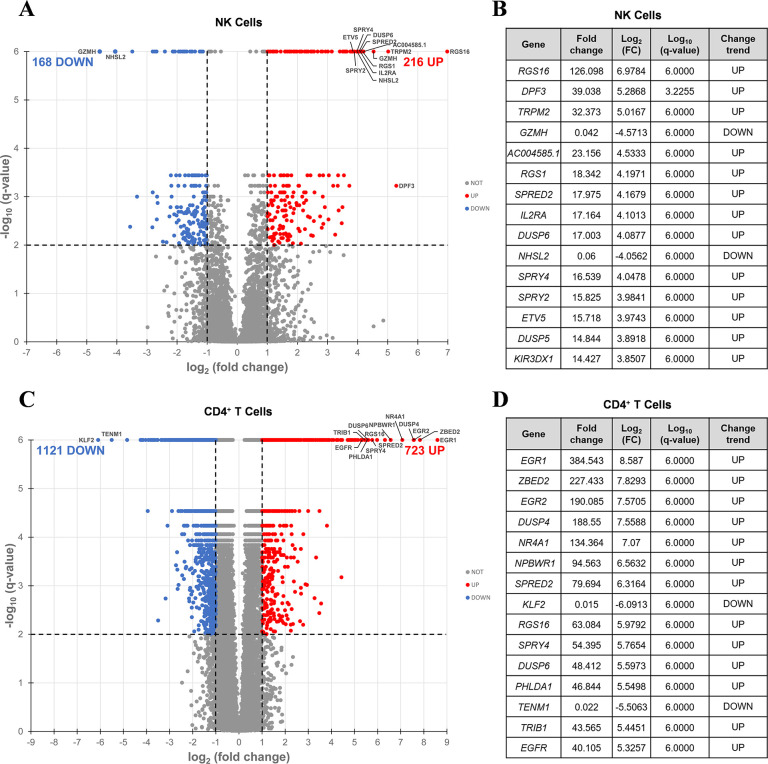
**Differentially expressed genes induced by SUW133 in NK and CD4^+^ T cells.** RNA-seq was performed on NK and CD4^+^ T cells cultured for 24 hours untreated (DMSO only) or with 10nM SUW133. Volcano plot of the distribution of all differentially expressed genes in NK cells (A) and CD4^+^ T cells (C). The red and blue dots represent the upregulated and downregulated genes (*q*-value < 0.01 and |log_2_FC| > 2), respectively. The 15 most differentially expressed genes are labeled on each plot and shown in tables (B) & (D).

Volcano plots were generated (Figure 4A and C, Supplemental Figure 4A and C, Supplemental Figure 5A and C), and based on these data, we identified the top 15 DEGs for each PKC modulator in both NK (Figure 4B, Supplemental Figure 4B, Supplemental Figure 5B) and CD4^+^ T cells (Figure 4D, Supplemental Figure 4D, Supplemental Figure 5D), the majority of which were upregulated. Amongst the top 15 DEGs, only 2 (*RSG1* and *RSG16*) were shared between all 3 PKC modulators in NK cells, while 10 (*DUSP4, EGR1, EGR2, NPBWR1, NR4A1, PHLDA1, RGS16, SPRED2, SPRY4, ZBED2*) were common to all 3 conditions in CD4^+^ T cells, suggesting that the effects of these drugs are more conserved in CD4^+^ T cells than in NK cells. *RSG16* was the only transcript upregulated by all PKC modulators in both cell types. *RSG16* inhibits G protein-coupled receptor signaling as well as cancer and inflammatory diseases through various other signaling pathways [92–94]. The magnitude of fold change for the top DEGs in all PKC treatment groups was lower in NK cells compared to CD4^+^ T cells, implying that treatment with PKC modulators more robustly perturbs the transcriptome of CD4^+^ T cells than NK cells. Additionally, similar to the ordinal ranking between each PKC treatment group in the number of DEGs, we saw that cells treated with prostratin had the highest magnitude fold change for gene expression, followed by SUW133, then bryostatin-1. Enrichment analysis of transcription factors using the transcriptional regulatory relationships unraveled by the sentence-based text-mining (TRRUST) database revealed several transcription factors responsible for the regulation of the observed phenotype (Supplemental Figure 6). PKC agonists are believed to induce latent HIV expression through NFκB signaling [37, 95–98]. Consistent with this, NFκB family transcription factors *NFKB1* and *RELA* were within the top 3 enriched transcription factors for all 3 PKC modulators in both NK and CD4^+^ T cells. Other transcription factors known to bind to the HIV-1 LTR pro-motor, such as *ETS1* and *SP1,* were also enriched.

### Bryostatin-1 Inhibits NK Cell Cytotoxic Activity

**Figure 5.**
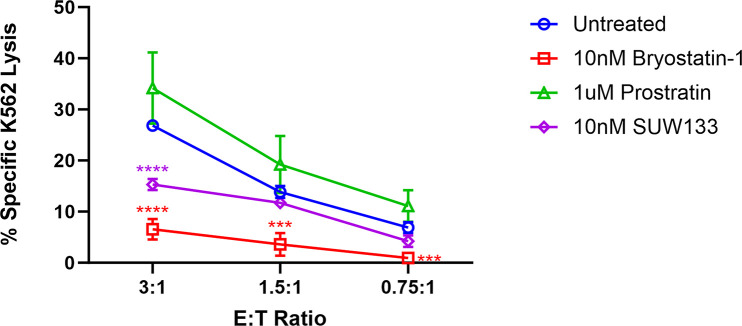
**Impact of PKC modulators on NK cell cytotoxicity.** NK cells were cultured for 24 hours untreated (DMSO only), with 10nM bryostatin-1, 1μM prostratin, or 10nM SUW133 and tested for cytotoxicity against K562 cells at the indicated effector-to target (E:T) ratios. The mean percentage specific lysis from 5 independent biological replicates was measured. Error bars indicate the standard error of the mean (SEM). An unpaired, unequal variance Student's *t*-test was performed, with (***) indicating *P* <0.001 and (****) indicating *P* < 0.0001.

Previous research examining the impact of PKC modulators on NK cell cytotoxicity suggested that exposure to prostratin increased NK cell-mediated killing, whereas it was diminished following exposure to bryostatin-1 [49, 99]. To further explore the functional effect of PKC modulators on NK cells and compare them with the effects of SUW133, we cultured NK cells for 24 hours in either bryostatin-1, prostratin, or SUW133 in parallel with untreated cells, then incubated in a 4-hour lysis assay with K562 cells, a human leukemia cell line that lacks HLA-antigen expression [100], at various effector-to-target (E:T) ratios (Figure 5). Confirming and extending the previously published findings, our results showed that pre-treatment of NK cells with bryostatin-1 consistently inhibited NK cell cytotoxicity while prostratin tended to enhance NK cell cytotoxic function, though the effects of prostratin were not statistically significant. SUW133, like bryostatin-1, decreased NK cell cytotoxicity, but this finding was only statistically significant at the highest tested E:T ratio (Figure 5). Because HIV infection results in changes that NK cells can recognize [49, 99], a similar experiment was conducted using HIV-infected CD4^+^ T cells, wherein NK cells were pre-treated for 24 hours in either bryostatin-1, prostratin, or SUW133 in parallel with untreated cells, then incubated in a 4-hour lysis assay with HIV-infected CD4^+^ T cells at an E:T ratio of 1:1 (Supplemental Figure 7). We observed statistically indistinguishable frequencies of total HIV-expressing (HA^+^) cells (Supplemental Figure 7A) and no difference in NK cell cytotoxicity between the untreated control and any of the PKC modulators (Supplemental Figure 7B). These data suggest that, although treatment of NK cells with PKC modulators resulted in increased expression of activation and degranulation markers, this did not result in increased killing by NK cells.

### Cytokines Released by NK Cells Treated with SUW133 Do Not Independently Reverse Latency in J-Lat Cell Lines

**Figure 6.**
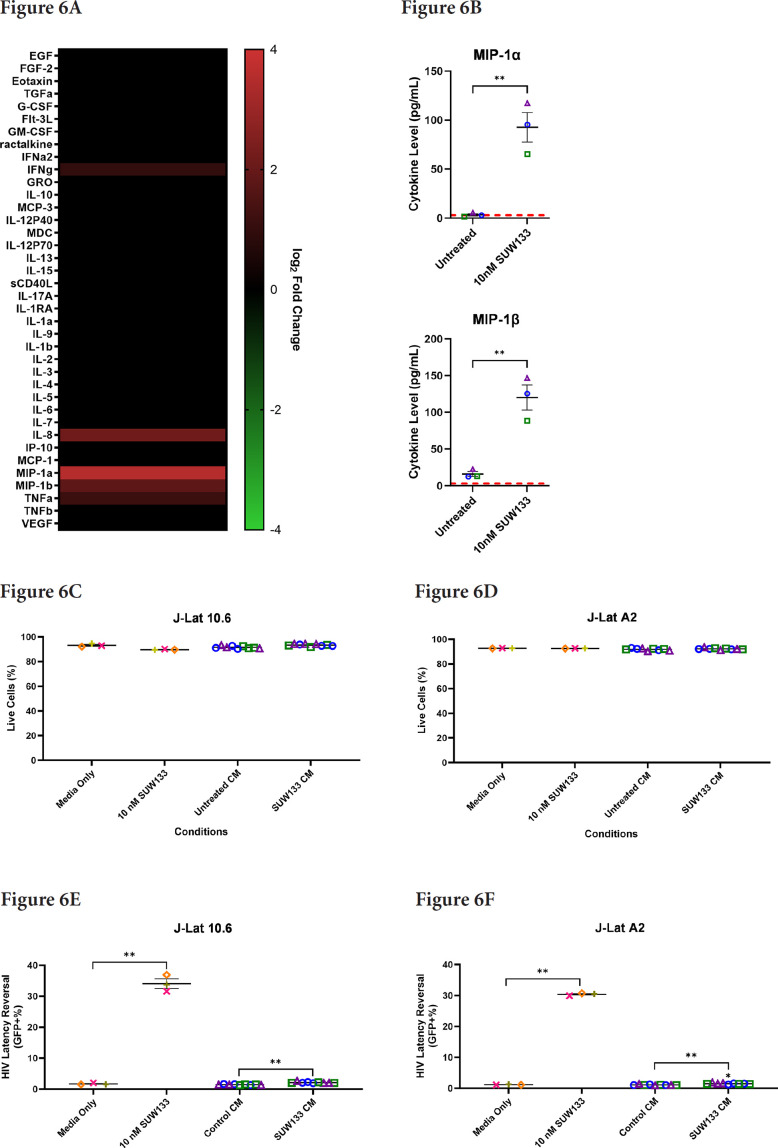
**Evaluation of secreted factors from NK cells treated with SUW133 and their effect on latency reversal in select J-Lat clones.** Conditioned media (CM) generated with NK cells from 3 different healthy human donors were analyzed using a Luminex 38-plex human cytokine immunoassay. (A) Heatmap showing the mean fold change relative to the untreated (DMSO only) control. (B) Example cytokine profiles from data shown in panel A, with each color and shape representing results from a different human donor. (C - F) Jurkat-Latency (J-Lat) clones (10.6 and A2) were treated with direct LRA or CM for 48 hours and assessed for viability (C, D) and GFP+ expression (E, F). All control conditions (media only and direct stimulations) were technical singlets in 3 independent biological replicates, resulting in an n = 3; For CM samples, technical triplicates in 3 independent biological replicates per donor (3 donors each) resulted in n = 9. A 2-tailed, unpaired, unequal variance Student's *t*-test was performed, with (*) indicating *P* <0.01 and (**) indicating *P* < 0.001. The single asterisk above the 10nM SUW133 CM condition in panel D corresponds to a comparison with direct stimulation with media only.

Cytokines such as IL-2, IL-7, and TNFα, as well as certain combinations of cytokines, have been shown to induce HIV latency reversal [101–104]. To assess whether cytokines produced by SUW133-stimulated NK cells could independently induce the expression of HIV in the absence of LRAs, NK cells were treated for 5 hours with SUW133, after which the LRA was removed, then the cells were washed and cultured in fresh media for an additional 24 hours to allow for the accumulation of cytokines. After 24 hours, LRA-free, cell-free supernatant, or conditioned media (CM), from stimulated cells was collected and analyzed for cytokine composition. Again, we observed higher levels of several cytokines compared to the control (Figure 6A), including MIP-1α and MIP-1β, though the measured concentrations (Figure 6B) were much lower than those seen with direct stimulation (Figure 2B), likely due to early removal of the compound.

Indirect latency reversal by CM was measured *in vitro* as previously described [59]. Briefly, select J-Lat clones were stimulated either directly with an LRA or with CM for 48 hours, then evaluated for viability and the expression of HIV via flow cytometry. J-Lat 10.6 cells contain a near-full-length HIV genome, while J-Lat A2 cells lack most HIV genes except the regulatory protein Tat, which enhances viral transcription [105, 106]. Both J-Lat clones contain a GFP reporter gene, which allows for the measurement of HIV expression. There was no significant difference in viability for either J-Lat clone between the different treatment groups and the control (Figure 6C and D). Consistent with previous studies [34, 48, 52, 107], we observed an increase in latency reversal when cells were directly stimulated with SUW133 compared to control (Figures 6E and F). Additionally, similar to what was observed with CM from LRA-stimulated PBMCs [48], there was little to no observed latency reversal by any of the CM samples in either latently infected cell line. However, there was a small (less than 1%) but statistically significant increase in GFP expression between the SUW133-treated CM compared to media only in J-Lat A2 cells, as well as between SUW133-treated CM and untreated CM in both J-Lat cell lines. This small (<1%) difference is unlikely to be biologically relevant and does not account for the robust LRA activity observed upon direct compound stimulation. Together, our findings suggest that the cytokines generated by NK cells stimulated with SUW133 do not independently stimulate latency reversal.

## DISCUSSION

Natural PKC modulators, such as bryostatin-1 and prostratin, can induce HIV transcription in latently infected cells [39, 41, 46, 47, 50, 107] and are under investigation as therapeutic agents against other diseases, including cancer [108, 109], Alzheimer's disease [110–112], and multiple sclerosis [113]. The scaled laboratory synthesis of bryostatin-1, now converted to a GMP synthesis, led to the production of synthetic bryostatin-1 analogs, some of which are more potent and less toxic than the parent compound [32, 48, 52–56]. One notable bryostatin-1 analog, SUW133, has been shown to successfully deplete latently infected cells and delay viral rebound in humanized mouse models [48]. Additionally and significantly, when combined with a kill agent (NK cells), SUW133 resulted in an even greater reduction in reservoir size and further delay in viral rebound [58]. While its direct effect on CD4^+^ T cells has been studied, it was not known whether SUW133 has any effect on NK cells.

In this study, we characterized the effect of SUW133 on primary human NK cells, in comparison to the classic PKC modulators bryostatin-1 and prostratin to determine whether it would result in increased NK function that may explain the previously observed results. We found that SUW133 induced increased CD69 and NKG2D expression, as well as increased CD107a expression, on NK cells, suggesting increased cellular activation and degranulation, respectively (Figure 1). Furthermore, the observed level of induction of these markers by SUW133 was similar to that induced by bryostatin-1 and prostratin, and there was no observed difference in viability between the different PKC treatment groups (Figure 1). Similarly, treatment of NK cells with SUW133 resulted in increased secretion of several pro-inflammatory cytokines, including IFNγ, MIP-1α, MIP-1β, and TNFα, at levels comparable to that of bryostatin-1 and prostratin (Figure 2). When comparing global transcriptomic changes induced by these PKC modulators on NK cells and CD4^+^ T cells, we observed distinct gene expression profiles (Figure 3A). All treatment groups had a greater effect in CD4^+^ T cells compared to NK cells, as evidenced by the quantity and magnitude of differentially expressed genes (Figure 3B, Figure 4, Supplemental Figure 4 and Supplemental Figure 5). The top 15 most differentially expressed genes in CD4^+^ T cells were more consistent amongst the 3 PKC modulators compared to NK cells, indicating a more conserved effect (Figure 4B and D, Supplemental Figure 4B and D, Supplemental Figure 5B and D). Consistent with prior studies [37, 95–98], transcription factor enrichment analysis showed that all PKC modulators resulted in responses consistent with enrichment for NFκB activity in both NK cells and CD4^+^ T cells (Supplemental Figure 6). When pre-treated with PKC modulators, then co-cultured with K562 target cells, NK cells exhibited different levels of cytotoxicity. At the tested concentrations and effector-to-target ratios, SUW133 and prostratin had fairly modest effects on NK cell cytotoxicity, while bryostatin-1 consistently inhibited killing (Figure 5). Moreover, when PKC modulator-treated NK cells were co-cultured with acutely infected HIV-infected CD4^+^ T cells, there was no observed difference in cytotoxicity (Supplemental Figure 7), further underscoring the fact that PKC modulators have limited effects on NK cell killing ability. To test whether NK cells had an indirect effect on latency reversal after PKC modulator stimulation, NK cells were exposed to SUW133, then the LRA was removed, and after further incubation, the conditioned media was harvested, profiled for cytokine composition, and tested for its capacity to induce latency reversal in cell lines. Although the CM containing secreted factors from SUW133-stimulated NK cells had elevated concentrations of various cytokines, little-to-no latency reversal was observed in both J-Lat 10.6 and A2 cells (Figure 6). It is important to note that although this study examines the direct effect of PKC modulators on NK cell cytotoxic function, we did not examine the effect that PKC modulators may have on other immune cells, such as dendritic cells, macrophages, and CD8^+^ T cells, which may affect NK cells indirectly. However, it has been shown previously that PBMCs stimulated with PKC modulators do not indirectly cause HIV latency reversal via cytokine expression [59]. Overall, our results, combined with our previous findings [38, 57, 58], suggest that the effects of PKC modulators on the eradication of the viral reservoir in the kick and kill approach is primarily due to increased provirus expression by CD4^+^ T cells, rather than an enhancement of NK cell effector function.

We suspect that the duration of NK cell exposure to the LRAs plays an important role in their cytotoxic function. In the previous *in vivo* humanized mouse study [58], we observed NK cells helped to decrease the viral reservoir when the initial injection of NK cells occurred 8 hours and 5 days after SUW133 exposure. Due to the short blood half-life and rapid absorption of PKC modulators *in vivo* [114, 115], it is likely that the NK cells had limited exposure to the LRA. All experiments performed in the current study were performed under prolonged exposure to LRAs (5–24 hours), yet only minor effects on NK function were observed, further supporting the interpretation that PKC modulators primarily augment the “kick” rather than the NK-mediated “kill” arm of this therapeutic approach.

An additional consideration is that the majority of experiments described here were performed in the absence of HIV infection and thus do not account for the possible contribution of the virus in augmenting the relationship between LRA-stimulated CD4^+^ T cells and NK cells in kick and kill approaches. For example, previous work has shown that the HIV accessory protein Vpr synergizes with prostratin to improve NK cell-mediated clearance of recently reactivated HIV-infected cells [49]. Expression of ULBP2, an NKG2D ligand, was increased on CD4^+^ T cells by both prostratin and HIV alone and further enhanced in combination, leading to sensitization of these cells to NK cell cytotoxicity [49]. While our transcriptomics data on uninfected cells also showed a modest increase in *ULBP2* by all PKC modulators (fold change = 1.294 for bryostatin-1, 1.335 for prostratin, and 1.486 by SUW133), this effect likely did not contribute to the observed enhancement of viral clearance by SUW133 + NK cells *in vivo*, as the virus used for infection, NL-HABC, lacks a functional Vpr protein [57]. Consistent with this hypothesis, we did not observe any difference in NK cell cytotoxicity when NK cells were pre-treated with PKC modulators and co-cultured with NL-HABC-infected CD4^+^ T cells (Supplemental Figure 7). Understanding the dynamics of other activating and inhibitory receptors induced by PKC modulators on both NK cells and Vpr-encoding HIV-infected CD4^+^ T cells requires further investigation. Furthermore, the effects observed in this study are based on *in vitro* assays, which may not accurately recapitulate what occurs in a clinical setting. Further *in vitro* evaluation of the efficacy of this kick and kill approach as a potential therapeutic strategy would involve related studies on cells from HIV-positive individuals.

Overall, this study improves our understanding of how PKC modulators affect NK cells in kick and kill strategies, indicating that their primary effects are related to augmenting the kick rather than kill arm of this therapeutic approach. Results from this study can be used to inform and optimize future HIV cure strategies as well as treatments for other diseases in which PKC modulators are employed.

## SUPPLEMENTARY DATA

Supplementary materials are available at the *Pathogens and Immunity* website. Supplementary data may be provided by the authors to benefit the reader. Supplementary data are not copyedited and are the sole responsibility of the authors. Questions or comments related to supplementary materials should be addressed to the corresponding author.
