# Supplementary material for: Defining the Effects of PKC Modulator HIV Latency-Reversing Agents on Natural Killer Cells
Source: Pathog Immun. 2024 Apr 24;9(1):108–37. doi: 10.20411/pai.v9i1.673 (PMC11101012; doi:10.20411/pai.v9i1.673)
Supplement: Supplementary Figures [file pai-9-108-s01.pdf]

PATHOGENS AND IMMUNITY
IN ASSOCIATION WITH CASE WESTERN RESERVE UNIVERSITY AND THE RICHARD J. FASENMYER FOUNDATION

SUPPLEMENTARY FIGURES

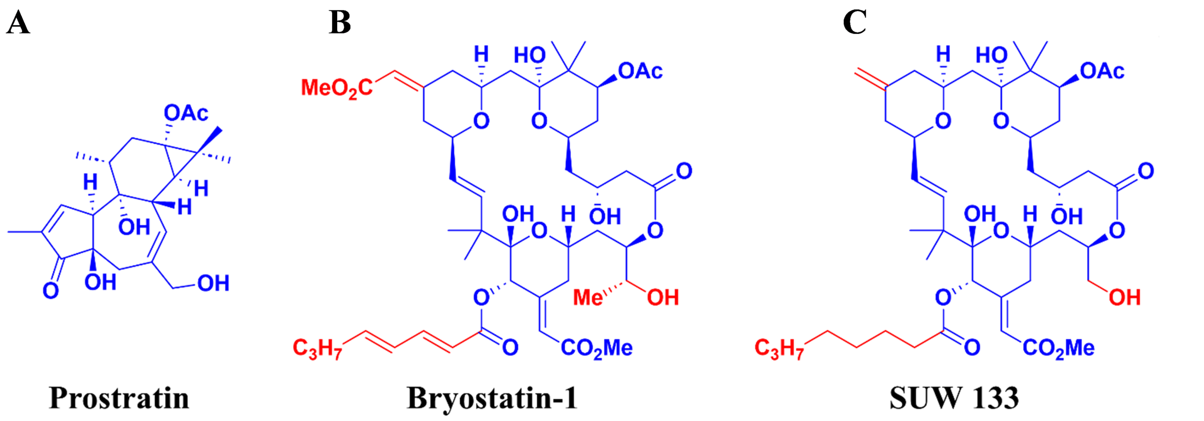

Supplementary Figure 1. Chemical structure of select PKC modulators. Chemical structures of prostratin (A), bryostatin-1 (B) and SUW133 (C). Moieties highlighted in red show the differences between bryostatin-1 and SUW133.

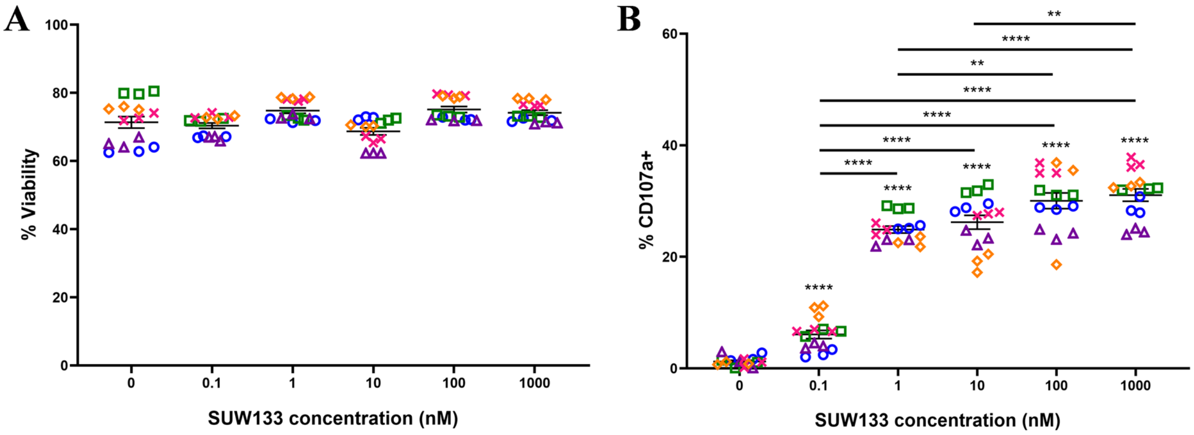

Supplementary Figure 2. Effect of SUW133 concentration on NK cell viability and degranulation. NK cells were cultured for 24 hours untreated (DMSO only) or with various concentrations of SUW133 and analyzed for viability (A) and degranulation, as measured by CD107a (B), via flow cytometry. All conditions were conducted in technical triplicates in 5 independent biological replicates (5 donors per condition) resulting in n = 15. Each bar graph depicts the mean with error bars indicating the standard error of the mean (SEM). A two-tailed, unpaired, unequal variance Student's *t*-Test was performed, with (**) indicating $p < 0.01$ and (****) indicating $p < 0.0001$. The asterisks above each bar graph in panel B correspond to a comparison with the untreated control.

Dimapasoc M, Moran JA, Cole SW, Ranjan A, Hourani R, Kim JT, Wender PA, Marsden MD, Zack JA. Defining the Effects of PKC Modulator HIV Latency-Reversing Agents on Natural Killer Cells. *Pathogens and Immunity*. 2024;9(1):108–137. doi: 10.20411/pai.v9i1.673

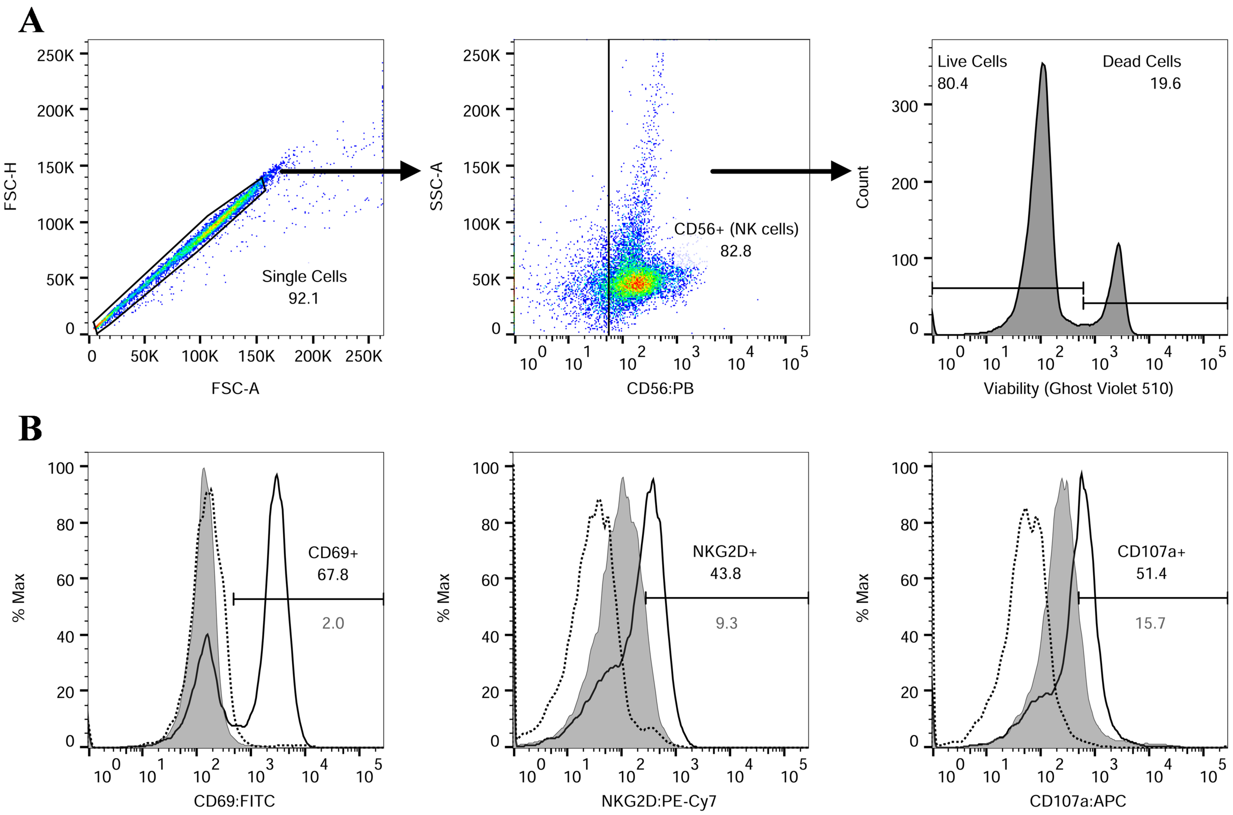

Supplementary Figure 3. Example flow cytometry profile/gating strategy related to Figure 1. NK cells were cultured for 24 hours untreated (DMSO only), with 10nM bryostatin-1, 1µM prostratin or 10nM SUW133 and analyzed for viability, CD69, NKG2D and CD107a via flow cytometry. (A) Representative scatter plots and histograms with percentages of gated cells shown. Positive gates were determined using an isotype control. (B) Population comparison of the frequency of CD69⁺, NKG2D⁺ CD107a⁺ NK cells among untreated (DMSO only; filled gray histograms, gray percentages) and treated cells (black lines, black percentages) along with the isotype control (dotted line) for a representative donor.

Dimapasoc M, Moran JA, Cole SW, Ranjan A, Hourani R, Kim JT, Wender PA, Marsden MD, Zack JA. Defining the Effects of PKC Modulator HIV Latency-Reversing Agents on Natural Killer Cells. *Pathogens and Immunity*. 2024;9(1):108–137. doi: 10.20411/pai.v9i1.673

A

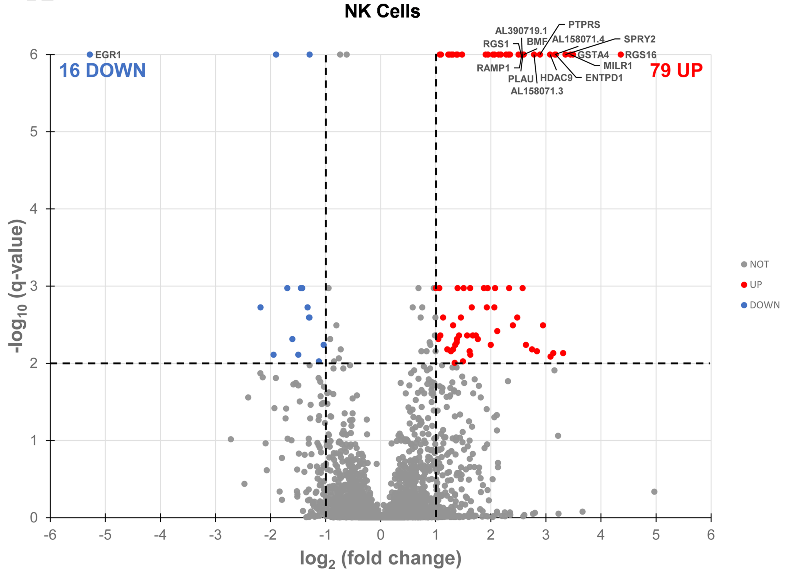

B

NK Cells

Gene	Fold change	Log_2 (FC)	Log_{10} (q-value)	Change trend
EGR1	0.026	-5.2815	6.0000	DOWN
RGS16	20.525	4.3593	6.0000	UP
GSTA4	11.25	3.4919	6.0000	UP
MILR1	10.906	3.4471	6.0000	UP
SPRY2	10.227	3.3543	6.0000	UP
ENTPD1	9.947	3.3143	2.1329	UP
AL158071.4	9.05	3.1779	6.0000	UP
HDAC9	8.955	3.1627	6.0000	UP
PTPRS	8.781	3.1344	2.1329	UP
AL158071.3	8.479	3.0839	2.0868	UP
BMF	8.433	3.076	6.0000	UP
PLAU	7.721	2.9487	2.4925	UP
AL390719.1	7.423	2.892	6.0000	UP
RAMP1	7.145	2.837	2.1574	UP
RGS1	6.895	2.7856	6.0000	UP

C

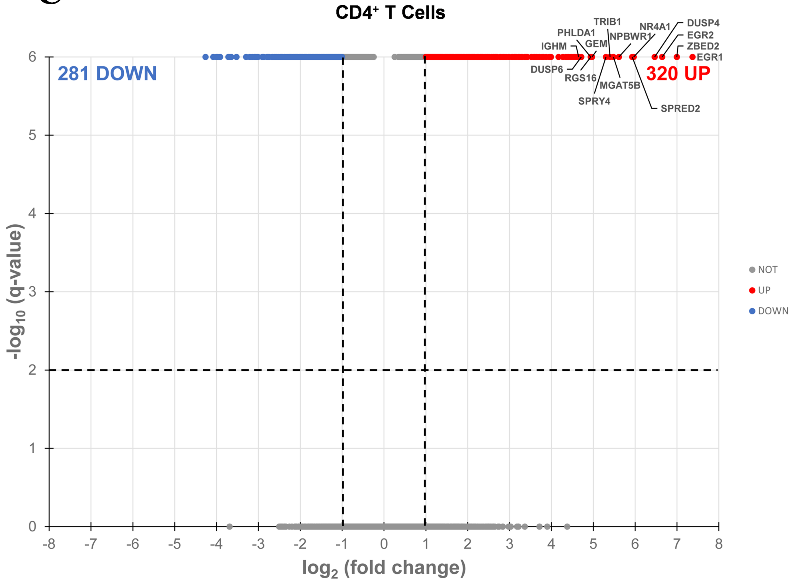

D

$CD4^+$ T Cells

Gene	Fold change	Log_2 (FC)	Log_{10} (q-value)	Change trend
EGR1	165.524	7.3709	1.00e-06	UP
ZBED2	127.566	6.9951	1.00e-06	UP
EGR2	100.024	6.6442	1.00e-06	UP
DUSP4	88.365	6.4654	1.00e-06	UP
NR4A1	62.475	5.9652	1.00e-06	UP
SPRED2	60.99	5.9305	1.00e-06	UP
NPBWR1	48.881	5.6112	1.00e-06	UP
MGAT5B	44.734	5.4833	1.00e-06	UP
TRIB1	42.242	5.4006	1.00e-06	UP
SPRY4	39.429	5.3012	1.00e-06	UP
GEM	31.333	4.9696	1.00e-06	UP
RGS16	30.762	4.9431	1.00e-06	UP
PHLDA1	30.631	4.9369	1.00e-06	UP
DUSP6	26.256	4.7146	1.00e-06	UP
IGHM	25.136	4.6517	1.00e-06	UP

Supplementary Figure 4. Differentially expressed genes induced by bryostatin-1 in NK and $CD4^+$ T cells. Volcano plot of the distribution of all differentially expressed genes in NK cells **(A)** and $CD4^+$ T cells (**C**) treated for 24 hours with 10nM bryostatin-1 compared to the untreated (DMSO only) control. The red and blue dots represent the upregulated and downregulated genes (*q*-value < 0.01 and $|log_2FC| > 2$), respectively. The 15 most differentially expressed genes are labeled on each plot and shown in tables (B) and (D).

Dimapasoc M, Moran JA, Cole SW, Ranjan A, Hourani R, Kim JT, Wender PA, Marsden MD, Zack JA. Defining the Effects of PKC Modulator HIV Latency-Reversing Agents on Natural Killer Cells. *Pathogens and Immunity*. 2024;9(1):108–137. doi: 10.20411/pai.v9i1.673

A

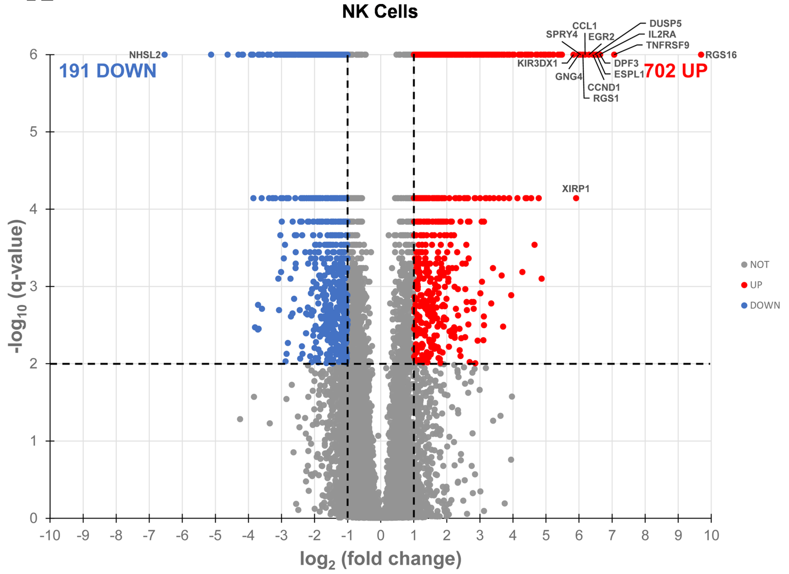

B

NK Cells

Gene	Fold change	Log_2 (FC)	Log_{10} (q-value)	Change trend
RGS16	827.204	9.6921	6.0000	UP
TNFRSF9	133.621	7.062	6.0000	UP
DPF3	99.685	6.6393	6.0000	UP
IL2RA	98.115	6.6164	6.0000	UP
ESPL1	92.732	6.535	6.0000	UP
NHSL2	0.011	-6.5344	6.0000	DOWN
DUSP5	88.641	6.4699	6.0000	UP
CCND1	85.568	6.419	6.0000	UP
EGR2	78.728	6.2988	6.0000	UP
CCL1	72.585	6.1816	6.0000	UP
RGS1	69.402	6.1169	6.0000	UP
SPRY4	66.019	6.0448	6.0000	UP
GNG4	63.589	5.9907	6.0000	UP
XIRP1	60.23	5.9124	4.1427	UP
KIR3DX1	56.965	5.832	6.0000	UP

C

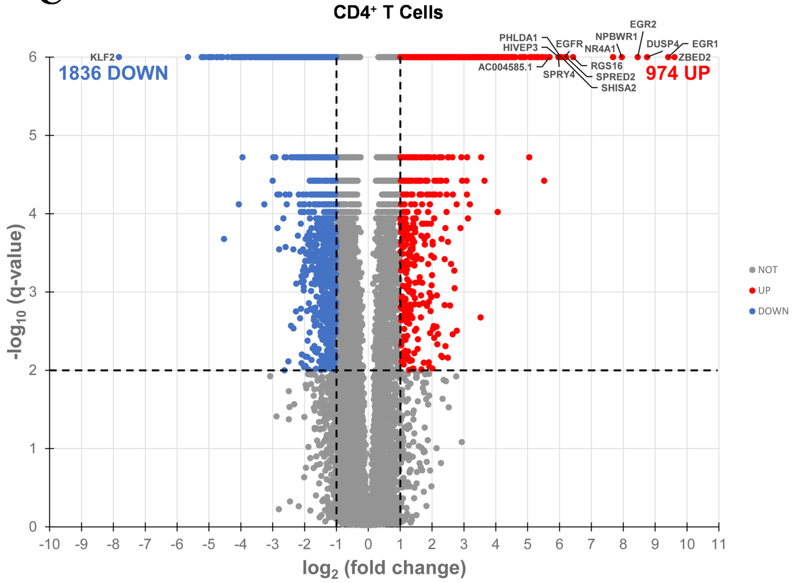

D

$CD4^+$ T Cells

Gene	Fold change	Log_2 (FC)	Log_{10} (q-value)	Change trend
ZBED2	779.767	9.6069	6.0000	UP
EGR1	680.334	9.4101	6.0000	UP
DUSP4	429.794	8.7475	6.0000	UP
EGR2	350.216	8.4521	6.0000	UP
NPBWR1	247.949	7.9539	6.0000	UP
KLF2	0.004	-7.8244	6.0000	DOWN
NR4A1	204.804	7.6781	6.0000	UP
RGS16	85.812	6.4231	6.0000	UP
SPRED2	73.874	6.207	6.0000	UP
EGFR	71.999	6.1699	6.0000	UP
SHISA2	66.741	6.0605	6.0000	UP
PHLDA1	65.676	6.0373	6.0000	UP
HIVEP3	62.866	5.9742	6.0000	UP
SPRY4	62.41	5.9637	6.0000	UP
AC004585.1	51.468	5.6856	6.0000	UP

Supplementary Figure 5. Differentially expressed genes induced by prostratin in NK and $CD4^+$ T cells. Volcano plot of the distribution of all differentially expressed genes in NK cells (A) and $CD4^+$ T cells (C) treated for 24 hours with 1µM prostratin compared to the untreated (DMSO only) control. The red and blue dots represent the upregulated and downregulated genes (q-value < 0.01 and $|\log_2FC| > 2$), respectively. The 15 most differentially expressed genes are labeled on each plot and shown in tables (B) and (D).

Dimapasoc M, Moran JA, Cole SW, Ranjan A, Hourani R, Kim JT, Wender PA, Marsden MD, Zack JA. Defining the Effects of PKC Modulator HIV Latency-Reversing Agents on Natural Killer Cells. *Pathogens and Immunity*. 2024;9(1):108–137. doi: 10.20411/pai.v9i1.673

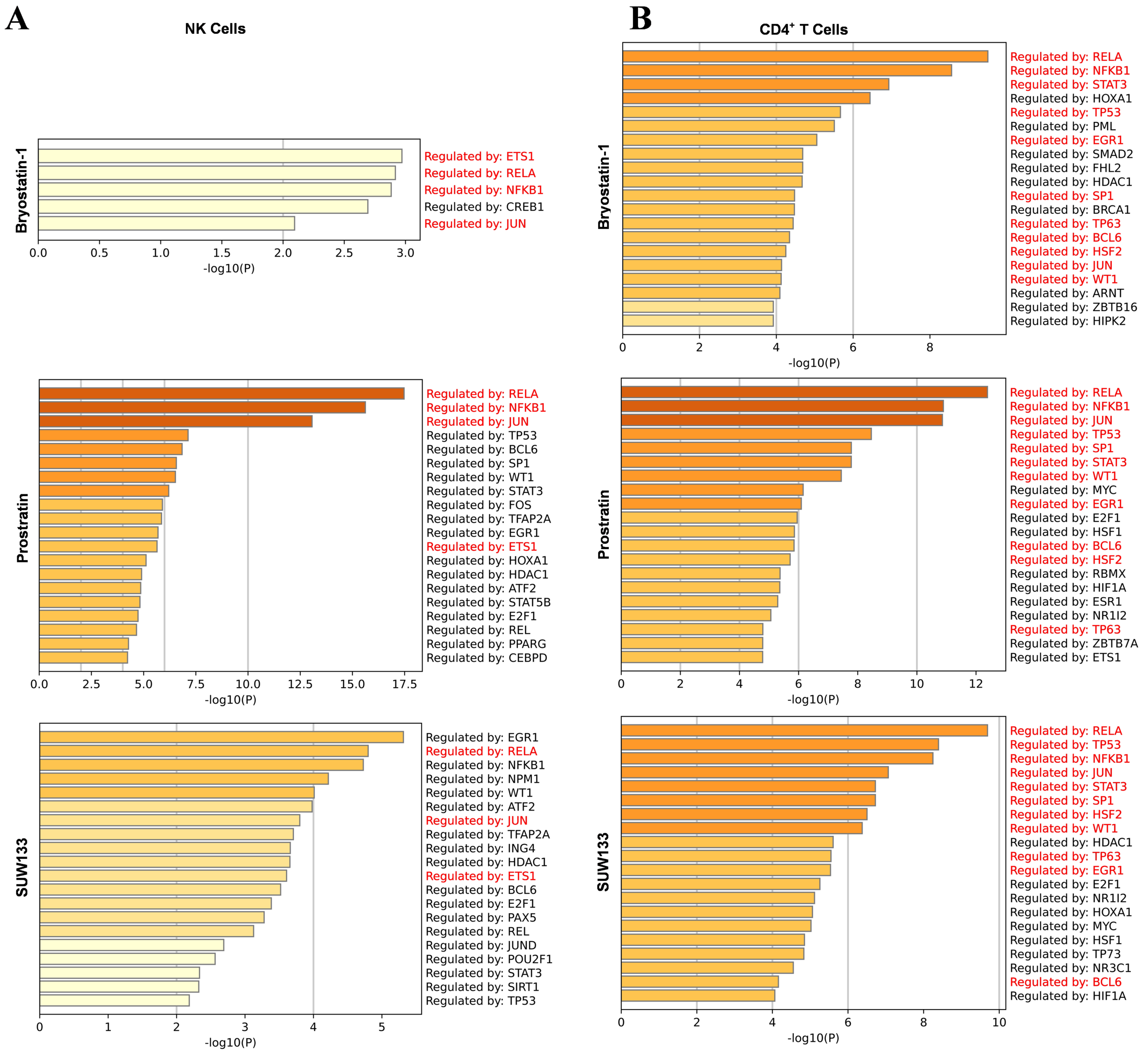

Supplementary Figure 6. Summary of enrichment of transcription factors for upregulated genes in NK and CD4+ T cells treated with PKC modulators. Metascape gene set enrichment analysis was performed on the lists of upregulated genes for NK cells (A) and CD4+ T cells (B) treated for 24 hours with 10nM bryostatin-1, 1µM prostratin or 10nM SUW133 using the transcriptional regulatory relationships unraveled by sentence-based text-mining (TRRUST) database. Terms with a $p < 0.01$, a minimum count of 3 and an enrichment factor >1.5 were collected and grouped into clusters based on their membership similarities. The p values were calculated based on the hypergeometric distribution. The data shown is ranked by statistical significance. Terms highlighted in red are shared between all PKC treatment groups.

PATHOGENS AND IMMUNITY
IN ASSOCIATION WITH CASE WESTERN RESERVE UNIVERSITY AND THE RICHARD J. FASENMYER FOUNDATION

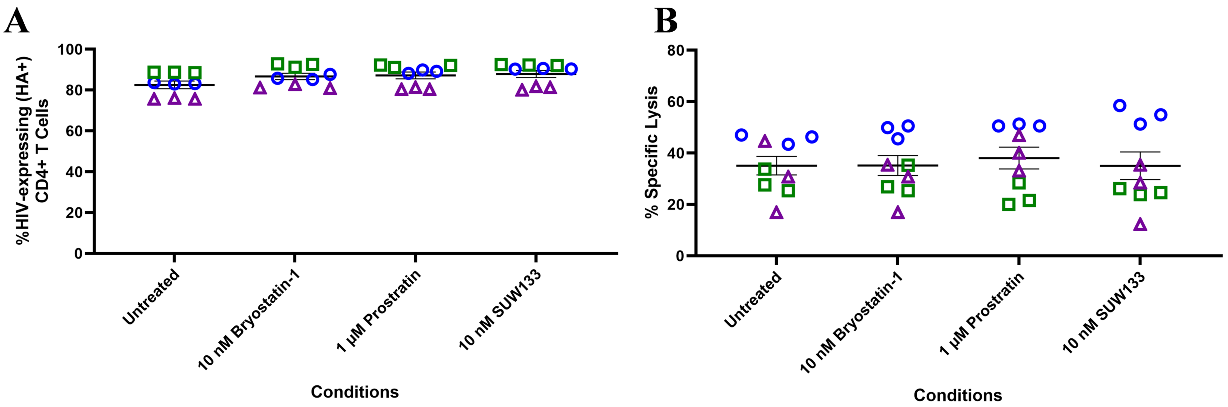

Supplementary Figure 7. Impact of PKC modulators on NK cell killing of HIV-infected CD4⁺ T cells. NK cells were pre-treated for 24 hours in vehicle control (DMSO only), with 10nM bryostatin-1, 1µM prostratin or 10nM SUW133 and co-cultured for 4 hours with HIV-infected $CD4^+$ T cells at a 1:1 effector-to-target (E:T) ratio. All conditions were conducted in technical triplicates in 3 independent biological replicates (3 donors per condition) resulting in n = 9. The percent HIV-expressing target cells (A) and specific lysis (B) was measured, with each color and shape representing results from a different human donor.

Dimapasoc M, Moran JA, Cole SW, Ranjan A, Hourani R, Kim JT, Wender PA, Marsden MD, Zack JA. Defining the Effects of PKC Modulator HIV Latency-Reversing Agents on Natural Killer Cells. *Pathogens and Immunity*. 2024;9(1):108–137. doi: 10.20411/pai.v9i1.673
